# Examining the Causal Inference of Leptin and Soluble Plasma Leptin Receptor Levels on Schizophrenia: A Mendelian Randomization Study

**DOI:** 10.3389/fpsyt.2021.753224

**Published:** 2021-10-27

**Authors:** Guoqing Chen, Qiuling Wang, Ranran Xue, Xia Liu, Hao Yu

**Affiliations:** ^1^Department of Psychiatry, Jining Medical University, Jining, China; ^2^Department of Psychiatry, Shandong Daizhuang Hospital, Jining, China

**Keywords:** schizophrenia, leptin, soluble plasma leptin receptor, genome-wide association study (GWAS), Mendelian randomization

## Abstract

**Background:** Observational studies that have supported the role of the leptin level in schizophrenia (SCZ) risk are conflicting. Therefore, we performed a two-sample Mendelian randomization (MR) analysis to investigate whether the circulating leptin and soluble plasma leptin receptor (sOB-R) levels play a causal role in SCZ risk.

**Methods:** We first selected five independent single-nucleotide polymorphisms (SNPs) associated with the circulating leptin level and three independent SNPs associated with the sOB-R level from two genome-wide association studies (GWASs) of European individuals. Then, we extracted their associations with SCZ using a large-scale GWAS that consisted of 40,675 patients with SCZ and 64,643 controls of European ancestry. We performed an MR analysis using the inverse variance-weighted (IVW) method to examine the causal effect of leptin on SCZ risk. Moreover, we performed sensitivity analyses to verify our MR results using the weighted median and MR-Egger methods.

**Results:** According to the IVW method, genetically predicted circulating leptin levels were not associated with SCZ risk (OR = 1.98, for per 1-SD unit increase in leptin level; 95% CI, 0.87–4.53; *p* = 0.10). In addition, the sOB-R level showed no causal effect on the SCZ risk using IVW (OR = 0.98 for per 1-SD unit increase in sOB-R level; 95% CI, 0.97–1.00; *p* = 0.06). Our sensitivity analysis results confirmed our MR findings.

**Conclusions:** By estimating the causal effect of leptin on SCZ risk using the MR methods, we identified no effect of genetically predicted circulating leptin or the sOB-R level on SCZ. As such, our study suggests that leptin might not be a risk factor for SCZ.

## Introduction

Schizophrenia (SCZ) is a major mental disease that represents a leading cause of impairment and burden worldwide (1). Cardiovascular diseases (CVDs) and metabolic syndrome (MetS) are highly prevalent in patients with SCZ, contributing a large extent to dramatically increased mortality (2–4). The high prevalence of CVDs in patients with SCZ may be partially attributed to unhealthy behaviors, including smoking, lack of physical activities, and dietary habits (5). More specifically, drug-naïve patients with SCZ are susceptible to metabolic disturbances (6). The underlying mechanisms of metabolic abnormalities in patients with SCZ are complicated and still not fully understood. Therefore, there is a great need to understand the mechanisms associated with metabolic abnormalities in SCZ patients.

Previous studies have revealed that SCZ patients showed a significantly increased level of excess adipose tissue (2). Among the adipokines, accumulating evidence has indicated that leptin may play a crucial role in both metabolic dysregulation and SCZ risk (7, 8). Leptin is known to act through the leptin receptor, and it is expressed in a variety of brain regions such as the cortex, hypothalamus, midbrain, and hindbrain (9). Leptin and its receptor are involved in the neuroendocrine and neurodevelopmental processes (10) and could modulate reward, mood, and neurological health (9). Several prospective studies have suggested that leptin and the sOB-R are associated with metabolic risk factors and might be able to predict adiposity and MetS (11, 12). Animal studies have suggested that leptin could modulate the activity of the dopaminergic and serotonergic systems, which are closely related to the pathogenesis of SCZ (13, 14). Several observational studies have indicated that leptin levels are altered in individuals with SCZ (15–18). However, the results of these observational studies have been conflicting, with reports of reduced (15) and increased leptin levels in patients with SCZ (16–18). However, the causal relationship between leptin and SCZ remains unclear. Moreover, these observational studies might have been affected by unmeasured confounding factors. Given the potential bias, observational studies cannot solely be relied upon for evidence (19). Generally, randomized controlled trials reduce potential bias in observational studies. To the best of our knowledge, no randomized controlled trials of leptin/sOB-R have been reported for SCZ. Given the high prevalence of metabolic abnormalities in patients with SCZ, it seems prudent to investigate whether leptin shows a causal role in SCZ risk.

Mendelian randomization (MR) was developed to investigate the causal relationship between exposure and the outcome of interest. This method overcomes the limitations of confounding factors and reverse causation in traditional studies (20). By using genetic variants as instrumental variables for a trait or disease, MR enables an investigation of associations independent of the conventional biases that accompany observational studies (20). MR approaches are particularly suitable for clarifying causal effects when observational studies are conflicting, and they provide useful intervention targets in randomized controlled trials. Recent genome-wide association studies (GWASs) have identified several common genetic variants that influence circulating leptin and sOB-R levels (21, 22), thus providing comprehensive data on the genetic determinants of leptin biomarkers. However, no MR analysis has been conducted to assess the casual relationship between leptin and SCZ risk.

In this study, by utilizing large-scale and GWAS datasets, we perform a two-sample MR study to examine the causal relationship between circulating leptin and sOB-R levels and SCZ.

## Materials and Methods

### Study Design

**Figure 1:**
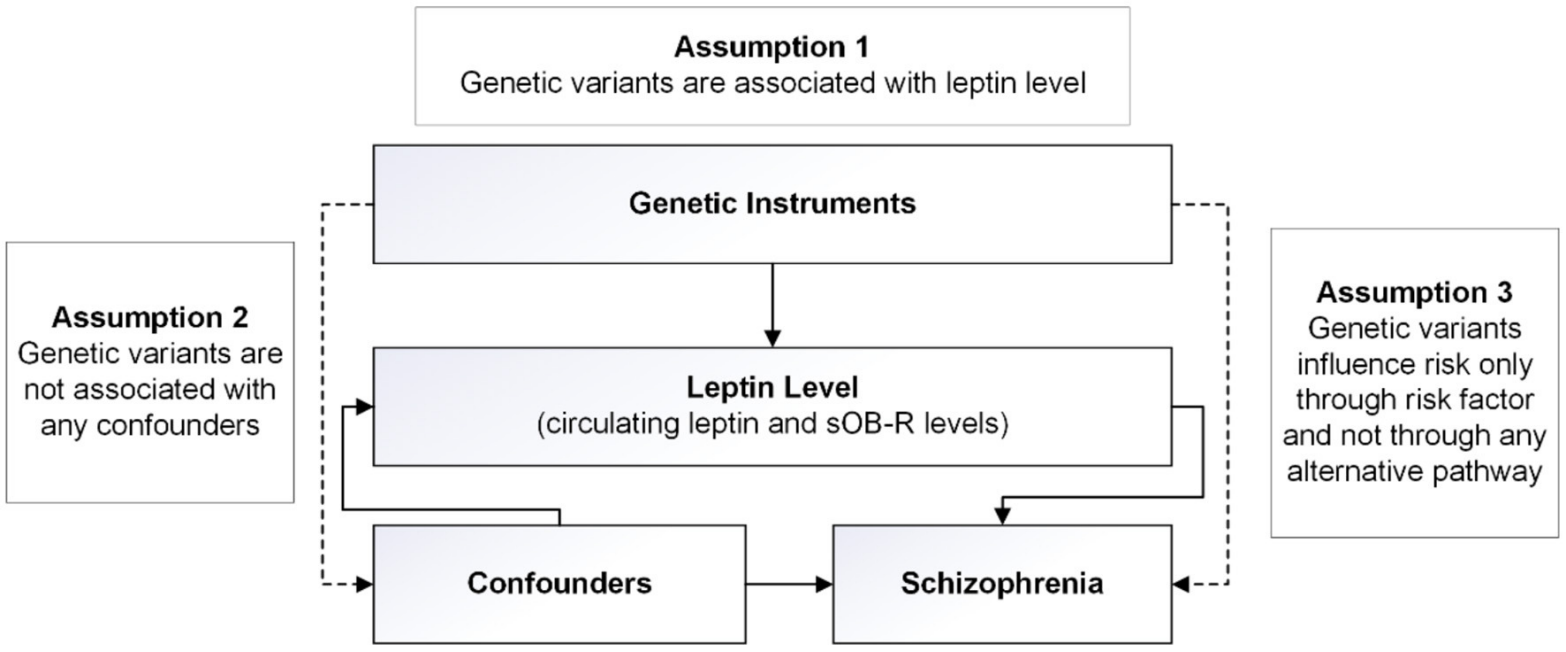
Design of the two-sample Mendelian randomization study. Three basic assumptions were as follows: (1) the single-nucleotide polymorphisms (SNPs) should be strongly associated with leptin level; (2) the SNPs should not be associated with confounders; and (3) the SNPs should not be directly associated with schizophrenia.

Our MR analysis was based on three core assumptions (Figure 1). First, the genetic variants used as instrumental variables for the leptin or sOB-R levels are associated with SCZ risk. Second, the genetic variants are not associated with any confounders. Third, the genetic variants are associated with SCZ through the leptin pathway and no other pathway.

### Data Source

We first obtained summary association results for two sets of genetic instruments: circulating leptin and sOB-R levels. For the leptin level, we derived summary data using a large-scale GWAS consisting of 32,161 individuals of European ancestry (22). Either plasma or serum leptin was measured in the morning or from fasting samples. Associations between genetic variants and the leptin level were investigated using a linear regression model, adjusting for age, age^2^, sex, body mass index (BMI), and any necessary study-specific covariates (22). For the sOB-R level, we identified genome-wide significant SNPs from a GWAS consisting of 1,504 individuals of European ancestry (21). The associations between the SNPs and the sOB-R level were adjusted for age and BMI in the original study (21). We then extracted summary associations between each genetic instrument and SCZ risk using large-scale SCZ GWAS data that consisted of 40,675 patients with SCZ and 64,643 healthy controls of European ancestry (23). More details regarding these GWAS can be found in the original studies (21–23).

### Instrumental Variable Selection

For the instrumental variables, we selected five single-nucleotide polymorphisms (SNPs) associated with the circulating leptin level and four SNPs associated with the sOB-R level at the genome-wide significance level (*p* < 5 × 10^−8^) (21, 22). We used linkage disequilibrium (LD) clumping to exclude the SNPs that had an *r*^2^ ≥ 0.01 with another SNP with a more significantly associated *p*-value in a 1-Mb window. The palindromic SNPs with ambiguous allele frequencies were removed from our MR analyses to eliminate potential biases (24). We then calculated the *F*-statistic that reflects the magnitude and the precision of the cumulative genetic effect for each SNP to estimate the strength for each SNP used in our MR analyses. The *F*-statistic was computed as follows: *F* = *beta*^2^/*se*^2^, where “*beta*” is the per-allele genetic effect on the SNP, and “*se*” represents the standard error of GX. To reduce the potential for weak instrument bias in the MR, we removed SNPs with *F*-statistics < 10 (25). We calculated the variance explained for a specific SNP using the following equation: variance explained = β^2^ × 2f × (1 – f), where β and f denote the effect of the SNP on the FT4 level and the minor allele frequency (MAF), respectively. The variance explained for a specific SNP is listed in Table 1. We then performed power calculations using the mRnd (https://shiny.cnsgenomics.com/mRnd/) (26) to test whether our study was adequately powered to detect clinically relevant changes in the SCZ risk.

**Table 1:** The characteristics of SNPs and their genetic associations with circulating leptin/sOB-R levels and schizophrenia.

**SNP**	**A1**	**A2**	**FRQ**	***F***	**Variance**	**beta.exposure**	**se.exposure**	**P.exposure**	**beta.outcome**	**se.outcome**	**pval.outcome**
**Circulating leptin level and schizophrenia**											
rs10487505	G	C	0.491	52.56	4.20E−04	0.029	0.004	1.99E−12	−0.0146	0.010	0.130
rs6071166	C	A	0.359	36.00	2.65E−04	0.024	0.004	1.75E−08	0.0413	0.010	3.04E−05
rs6738627	A	G	0.412	25.00	1.94E−04	0.020	0.004	1.92E−06	0.0004	0.010	0.970
rs780093	C	T	0.589	36.00	2.79E−04	0.024	0.004	3.80E−10	0.0004	0.010	0.970
rs900400	T	C	0.610	27.56	2.10E−04	0.021	0.004	1.17E−07	0.0173	0.010	0.080
**sOB-R level and schizophrenia**											
rs17412403	T	C	0.530	58.18	5.36E−02	0.328	0.043	8.66E−14	−0.0102	0.010	0.296
rs17415296	C	A	0.844	1799.82	5.16E−01	1.400	0.033	1.00E−200	−0.0172	0.013	0.179
rs4655537	A	G	0.364	63.27	5.67E−02	0.350	0.044	7.42E−15	−0.0131	0.010	0.193

*We calculate F-statistic using the following formula: F = beta^2^/se^2^. We calculated the variance explained for a specific SNP using the equation beta^2^ × 2f × (1 – f), where f denotes the minor allele frequency (MAF)*.

*SNP, single-nucleotide polymorphism; A1/A2, reference allele/alternative allele; beta.exposure, beta value in genome-wide association study (GWAS) of exposure; se.exposure, standard error of beta value for exposure; beta.outcome, beta value in GWAS of outcome; se.outcome, standard error of beta value for outcome*.

### Mendelian Randomization Analysis

For the SNP, we calculated the MR estimate using the Wald estimator of exposure and outcome summary statistics with the standard error using the delta method (27). We then combined the MR estimates of individual SNPs using the inverse-variance-weighted fixed-effect (IVW_FE) model (28). We considered a two-sided *p* < 0.05 to be significant. Because the MR results might be confounded when the genetic instruments show horizontal pleiotropy, we compared the IVW results with the weighed median (29) and the MR-Egger (24) tests, whose estimates are known to be relatively robust to horizontal pleiotropy (30). Using Cochran's *Q*-test, we examined the heterogeneity produced by multiple genetic variants in the IVW analyses (31). If Cochran's *Q*-test suggested potential heterogeneity, we used the IVW random-effects model (IVW_RE) to assess the association between leptin and SCZ (31). By removing one genetic instrument at a time, we performed the leave-one-out analysis to identify genetic variants with exaggerated effects on the overall estimate (32). We used the MR-Pleiotropy RESidual Sum and Outlier (MR-PRESSO) method to detect the SNP outliers (33). If the SNP outliers were identified, we removed them to correct for possible pleiotropic effects and repeated the MR analysis. All of the MR analyses were performed using the R package “TwoSampleMR” (32).

## Results

### Circulating Leptin Level and Schizophrenia

**Figure 2:**
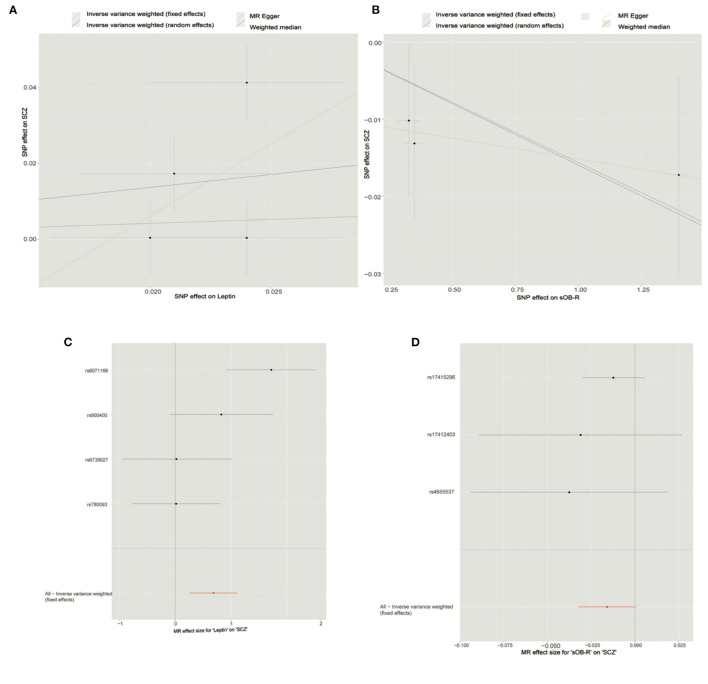
Mendelian randomization analysis showing the effects of circulating leptin and soluble leptin receptor (sOB-R) levels on schizophrenia (SCZ) risk. **(A)** Mendelian randomization (MR) analyses of circulating leptin and SCZ. **(B)** MR analyses of sOB-R level and SCZ. **(C)** Forest plot for MR analyses of circulating leptin and SCZ. **(D)** Forest plot for MR analyses of sOB-R level and SCZ.

After LD-based clumping, five independent SNPs were not in the LD (defined as *r*^2^ < 0.2, windows 100 kb) with other genetic variants for the circulating leptin level. The associations between each SNP with the circulating leptin level and the SCZ risk are listed in Table 1. For the five SNPs associated with the circulating leptin level, we removed the SNP (rs10487505) for being palindromic with intermediate allele frequencies. Using the four retained SNPs, we found that the genetically predicted leptin level had a significant effect on the SCZ risk (IVW_FE: OR = 1.98, for per 1-SD unit increase in leptin level; 95% CI, 1.29–3.06; *p* = 1.97E−03; Figure 2; Table 2). Our sensitivity analysis suggested that the weighed median (OR = 1.69, *p* = 0.11) and MR-Egger (OR = 5.33, *p* = 0.78) provided consistent results with the IVW analysis, but with less precision (Figure 2). However, we detected significant heterogeneity using Cochran's *Q*-test (*Q* = 10.87, *p* = 0.01). Therefore, we moved to the IVW random model and found no significant effect on the SCZ risk (IVW_RE: OR = 1.98, for per 1-SD unit increase in leptin level; 95% CI, 0.87–4.53; *p* = 0.10; Figure 2; Table 2). Additionally, we detected no SNP outlier using the MR-PRESSO test (*p* > 0.05) and no pleiotropy when using the MR-Egger method (intercept = −0.07, se = 0.14, *p* = 0.66). We plotted the leave-one-out plots in the MR analyses (Supplementary Figure S1). Based on a sample size of 105,318 individuals (40,675 patients with SCZ and 64,643 healthy controls) and setting alpha to 0.05 and the variance explained to 0.1%, our study had a power of 89% power to detect effects on SCZ as an OR of 1.98 per 1-SD change in the leptin level on the log scale.

**Table 2:** Two-sample MR estimates of relationship between genetically predicted circulating leptin/sOB-R levels and SCZ.

**Exposure**	**Method**	***N***	**OR**	***p***
Leptin	IVW_FE	4	1.98	1.97E−03
Leptin	IVE_RE	4	1.98	0.10
Leptin	MR Egger	4	44.8	0.56
Leptin	Weighted median	4	1.24	0.49
sOB-R	IVW_FE	3	0.98	0.06
sOB-R	MR Egger	3	0.99	0.76
sOB-R	Weighted median	3	0.98	0.08

*In this study, we performed MR analyses using circulating leptin/sOB-R levels as exposure and SCZ as outcome*.

*OR, odds ratio; N, number of single-nucleotide polymorphisms (SNPs) used in the MR analyses; IVW_FE, inverse-variance-weighted (IVW) fixed-effects model; IVW_RE, inverse-variance-weighted (IVW) random-effects model; MR, Mendelian randomization; sOB-R, soluble leptin receptor; SCZ, schizophrenia*.

### The Soluble Leptin Receptor Level and Schizophrenia

By using LD-based clumping, we removed the SNP (rs1137100) that was in the LD with other genetic variants for the sOB-R level. We list the associations between each SNP with the sOB-R level and SCZ risk in Table 1. By using the three retained SNPs, we found that the genetically predicted sOB-R level had no significant effect on the SCZ risk (IVW_FE: OR = 0.98 for per 1-SD unit increase in sOB-R level; 95% CI, 0.97–1.00; *p* = 0.06; Figure 2; Table 2). Our sensitivity analysis suggested that the weighed median (OR = 0.98, *p* = 0.08) and MR-Egger (OR = 0.99, *p* = 0.76) methods provided consistent results with the IVW analysis, but with less precision (Figure 2). For the four SNPs associated with the circulating leptin level, we detected no pleiotropy or heterogeneity using Cochran's *Q-*test (*Q* = 0.98, *p* = 0.61) and MR-Egger (intercept = −0.01; se = 0.01; *p* = 0.51). We detected no SNP outlier using the MR-PRESSO test (*p* > 0.05). The leave-one-out plot is shown in the MR analyses (Supplementary Figure S2). By using mRnd software, we calculated that our study had a power of 71% power to detect effects on SCZ as an OR of 0.98 per 1-SD change in leptin level on the log scale.

## Discussion

This MR study investigated the causal relationship between leptin and SCZ, and no causal relationship between the circulating leptin level and SCZ was identified. This was consistent with a previous observational study (34). In addition, we found no causal effect of the sOB-R level on SCZ risk. Given that sOB-R is a leptin binding protein, the sOB-R had an inverse relationship with the circulating leptin levels. Therefore, these consistent findings further supported a lack of a causal effect of leptin on SCZ. Our results from the weighted median and MR-Egger methods confirmed the robustness of this lack of causal effect, as the results were generally similar to the primary analysis that utilized IVW methods. Therefore, our study suggests that leptin and sOB-R levels are unlikely to affect SCZ risk.

Our study provided new clues for the relationship between leptin levels and SCZ risk. The accumulated evidence suggested that leptin levels may be involved in SCZ risk. Observational studies and meta-analysis have suggested that leptin levels are elevated in patients with SCZ (35, 36). A longitudinal study found that antipsychotic-naïve patients with SCZ had decreased leptin levels than healthy controls at baseline (15). In addition, there are few observational studies that have reported no significant association between leptin and SCZ (34). However, whether these observed associations are causal is still unclear because the results in observational studies are susceptible to reverse causality and unknown confounding factors. For example, some studies have shown that leptin levels are elevated, not only in patients taking atypical antipsychotics (37) but also in drug-free (38) and drug-naïve subjects (39). Unfortunately, few studies have examined the association between the sOB-R level and SCZ. Therefore, new methods are needed to assess the causal relationship between leptin and SCZ. By utilizing genetic variants as proxies for exposure, MR analyses can provide an unbiased estimate of association with the outcome of interest. In this study, we detected no evidence using the MR approach that leptin and the sOB-R levels are associated with SCZ risk. To our best knowledge, this is the first study with a sufficient sample size under the MR assumptions to examine whether there is a causal association between the leptin/sOB-R level and SCZ risk.

Our study is subject to several limitations. First, leptin levels might be affected by many factors. Recent observational studies have suggested that leptin levels are associated with demographic (age, sex, and ethnicity), anthropometric measures (body mass index, skinfold thicknesses, waist and hip circumferences, waist/hip ratio, total body water, fat-free mass, and fat mass) (40, 41), and medication history. The GWAS summary data of the leptin levels used in our MR analysis were adjusted for age, age^2^, sex, BMI, and any necessary study-specific covariates (22); and the selected significant SNPs for the sOB-R levels were adjusted for BMI (21). This might have reduced the potential bias. Moreover, our sensitivity analysis resulted in effect estimates with similar patterns and corroborated the primary MR results. However, the GWAS samples of the circulating leptin and the sOB-R level consisted of a large proportion of middle-aged individuals (21, 22). Thus, it is still unknown to what extent these data accounted for early life exposures that might be involved in the development of SCZ. Additionally, we performed the MR analysis using the GWAS summary data and could not conduct stratified analyses with covariates, such as medication history, physical/dietary habits, and substance use. Therefore, future GWAS or MR analyses should consider these factors when assessing the role of leptin and sOB-R levels. Second, our MR analyses focusing on the European population and generalizability could not be assumed. Third, the sample size and the number of variants for analysis were relatively small. We found no evidence for a causal relationship between leptin and sOB-R levels and SCZ risk. Further research is necessary to understand the pathways underlying the association and resolve whether the leptin level is a useful predictor of SCZ risk that can help guide therapeutic interventions.

In conclusion, we found no significant causal relationship for circulating leptin or sOB-R level on SCZ risk. Overall, our results did not support the hypothesis that increased leptin or sOB-R level is a risk factor for SCZ.

## Data Availability Statement

The original contributions presented in the study are included in the article/Supplementary Material, further inquiries can be directed to the corresponding author.

## Author Contributions

HY designed the study and contributed to the analysis and interpretation of data. GC, RX, QW, and HY did the statistical analyses and prepared the tables and figures. GC and HY wrote the first draft of the manuscript. GC provided further data interpretation. All authors contributed to drafting the work or revising it critically for important intellectual content, made substantial contributions to the concept and design of the study and acquisition, analysis, and interpretation of data.

## Funding

This study was funded by the National Natural Science Foundation of China (81901358), Medical and Health Science and Technology Development Plan of Shandong Province (2014WS0278 and 2018WS457), and Young Taishan Scholars of Shandong Province (tsqn201909146). The funders had no role in the design and conduction of this study.

## Conflict of Interest

The authors declare that the research was conducted in the absence of any commercial or financial relationships that could be construed as a potential conflict of interest.

## Publisher's Note

All claims expressed in this article are solely those of the authors and do not necessarily represent those of their affiliated organizations, or those of the publisher, the editors and the reviewers. Any product that may be evaluated in this article, or claim that may be made by its manufacturer, is not guaranteed or endorsed by the publisher.
