# Supplementary material for: Examining the Causal Inference of Leptin and Soluble Plasma Leptin Receptor Levels on Schizophrenia: A Mendelian Randomization Study
Source: Front Psychiatry. 2021 Oct 27;12:753224. doi: 10.3389/fpsyt.2021.753224 (PMC8578685; doi:10.3389/fpsyt.2021.753224)

**Supplementary Figure 1.** **Plot from leave-one-out sensitivity analysis for the instrument variable set of leptin level.** The solid lines represent 95% confidence intervals.


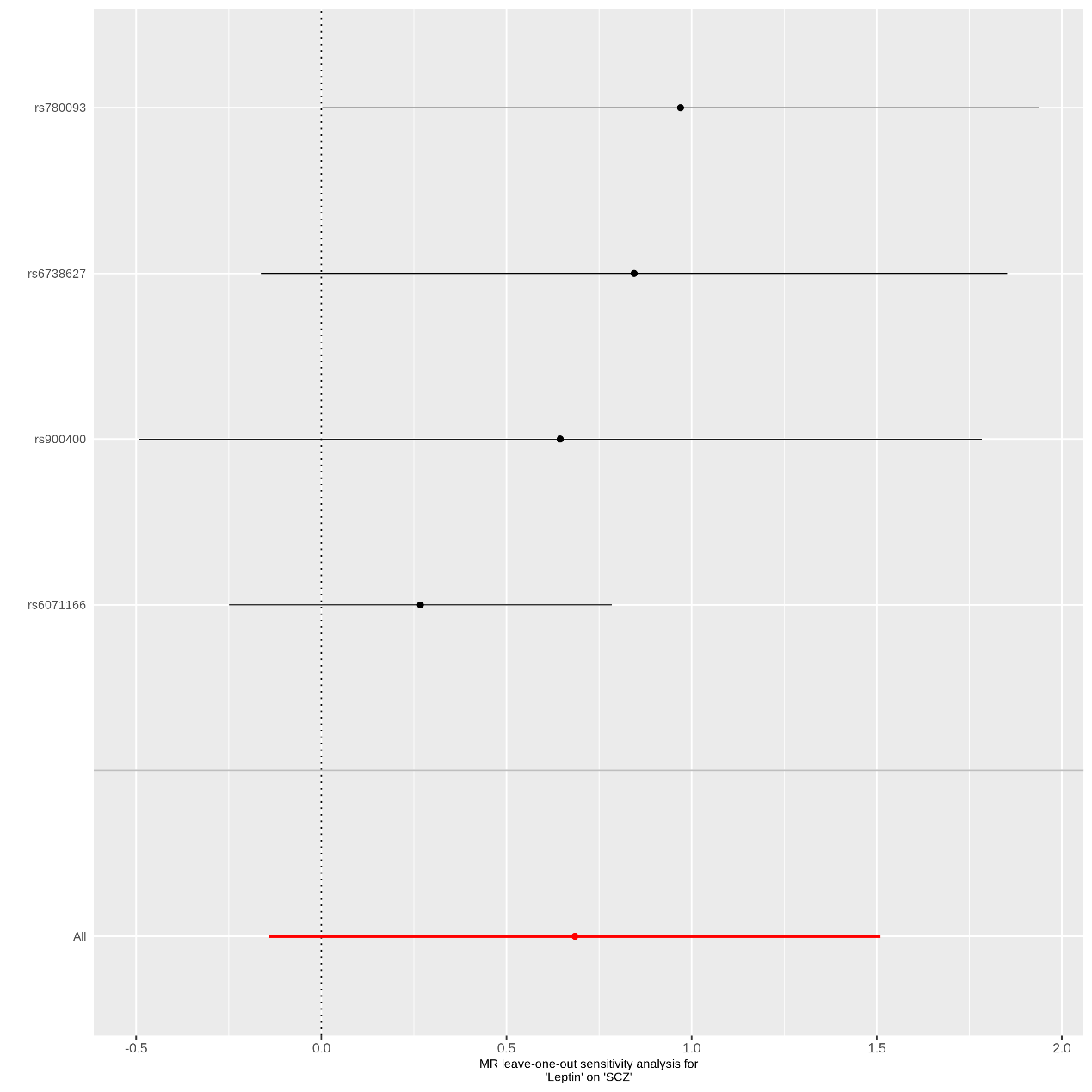


**Supplementary Figure 2.** **Plot from leave-one-out sensitivity analysis for the instrument variable set of sOB-R level.** The solid lines represent 95% confidence intervals.


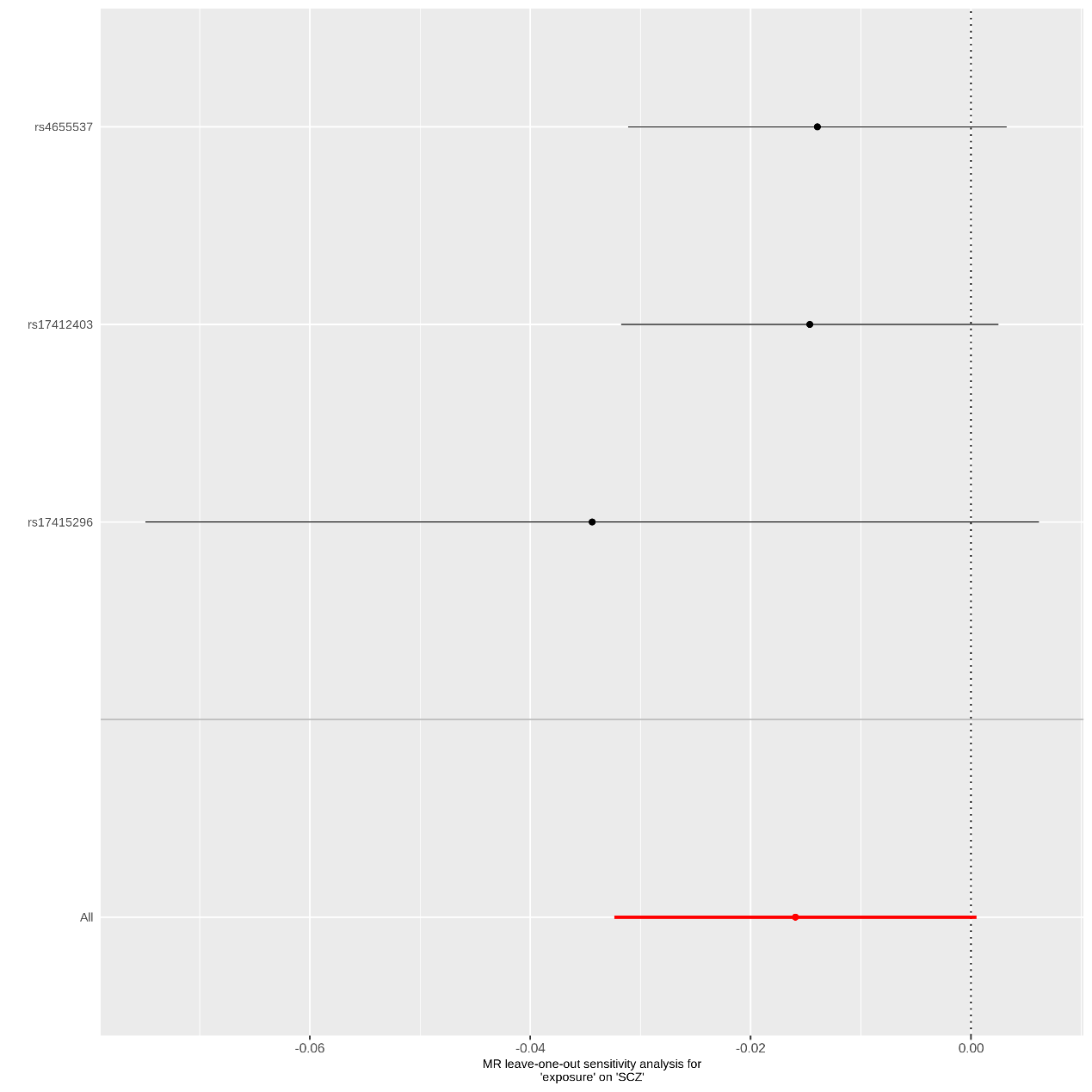

Supplement: Supplementary file 1 [file Data_Sheet_1.docx]
